# Skating

**Published:** 1873-01

**Authors:** 


### SKATING.

To skate safely, healthily and enjoyably, the following suggestions should be attended to:

1. The best pattern of a skate is that which does not require any heel plate or key or pernicious strap which, by impeding the circulation of the blood, endangers freezing, mortification, and the loss of the limb. The latest and best device attaches the skate to the sole of the foot, not with a key, but an ingeniously attached lever, which is almost instantly adjusted ; ladies especially should use this skate.

2. It is better, if the distance is not over a mile, to walk to the skating place ; this prevents one from getting chilled before the skating commences.

3. If possible, walk home so as to keep up the circulation ; for after the skating is over there is more or less of a perspiration, and to sit still in a vehicle, especially if it is an open one, involves the certainty of cooling off too quick, causing a bad cold, if not an attack of inflammation of the lungs, or frost-bitten feet.

4. Go to the skating with an extra coat or shawl, to be laid aside while skating, and to be resumed the moment the skating is over, even before the skates are taken off.

5. While skating, keep in motion ; or rest but a minute or two at a time, for if the wind is blowing a longer rest endangers a chill.

6. By all means skate in moderation, which prevents over-heating and over-fatigue, which, especially when combined, leave the system exceedingly susceptible of a chill.

7. Very few, especially ladies, should remain on the ice longer than an hour at a time.

8. If one falls or breaks into the water and assistance should be required, the person giving aid should lay flat on the ice and extend the hand over the edge ; or better, if a board or fence rail could be had, slide it along to the sufferer ; let him catch hold with both hands, and allow himself to be drawn out on all fours—not attempting to get on the ice again on his knees and elbows, but spread his weight over as large a surface as possible. It is safer still if a third person lays flat on the ice and catches hold of the ankle of the one in advance of him in case he too should break in. These things should be taught to children early, for they are most liable to be found in need of aiding one another. A little girl of twelve recently saved one of her companions in the way above described, she having heard her parents speak of it at some previous time.

9. Let it be arranged beforehand that when the skaters return home they shall be welcomed to a warm room, a blazing fire, and a hot lunch or supper of stewed oysters, "chicken fixings and things."

